# Fatal blunt chest trauma: an evaluation of rib fracture patterns and age

**DOI:** 10.1007/s00414-022-02866-2

**Published:** 2022-07-14

**Authors:** Siobhan O’Donovan, Corinna van den Heuvel, Matthew Baldock, Melissa A. Humphries, Roger W. Byard

**Affiliations:** 1 School of Biomedicine, The University of Adelaide, Helen Mayo North, Level 2, Room N207 Frome Road, SK 5005 Adelaide, Australia; 2 Forensic Science SA, Adelaide, Australia; 3 Centre for Automotive Safety Research, The University of Adelaide, Adelaide, Australia; 4 School of Mathematical Sciences, The University of Adelaide, Adelaide, SA Australia

**Keywords:** Rib fractures, Fatal vehicle crashes, Age, Injury, Forensic

## Abstract

The following study was undertaken to determine if any specific occupant characteristics, crash factors, or associated injuries identified at autopsy could predict the occurrence or number of fractured ribs in adults. Data were accrued from the Traffic Accident Reporting System (TARS) and coronial autopsy reports from Forensic Science SA, Adelaide, South Australia, from January 2000 to December 2020. A total of 1475 motor vehicle fatalities were recorded in TARS between January 2000 and December 2020, and 1082 coronial autopsy reports were identified that corresponded to TARS fatal crash data. After applying exclusion criteria involving missing data, 874 cases were included in the analysis. Of the 874 cases, 685 cases had one or more rib fractures. The leading cause of death for those with rib fractures was multiple trauma (54%), followed by head injury (17%) and chest injuries (10%). The strongest predictor of one or more rib fractures was increasing age (*p* < 0.001). Other factors found in the regression to be predictive of the number of rib fractures were the presence of a variety of other injuries including thoracic spinal fracture, lower right extremity fracture, splenic injury, liver injury, pelvic fracture, aortic injury, lung laceration, and hemothorax. Age is most likely associated with increasing rib fractures due to reduced tolerance to chest deflection with greater injuries occurring at lower magnitudes of impact. The association of other injuries with rib fractures may be a marker of higher impact severity crashes.

**Supplementary Information:**

The online version contains supplementary material available at 10.1007/s00414-022-02866-2.

## Introduction

Blunt force chest injuries with associated rib fractures are frequent in motor vehicle crashes. Injuries to the thorax can occur from impacts with the steering wheel or instrument panel in frontal crashes, from side doors in side impact crashes, or from a combination of these. Fractured ribs may lead to injury of thoracic organs, particularly with the sharp ends of the ribs having the potential to tear/puncture vessels and the lungs, or the pleura leading to pneumothorax or hemothorax [1, 2].

Cadaveric studies and finite element models are often used to simulate and predict rib fracture patterns in injury research, and studies have used clinical data to identify patterns of rib fracture occurring with multiple causes of trauma including motor vehicle crashes [3–8].

The following study evaluates the patterns of rib fracture in adult occupants involved in fatal motor vehicle crashes and combines coronial autopsy report injury descriptions with crash data information. Seat belt wearing is mandatory for all motor vehicle occupants in Australia. The aims of this study are to determine if any specific occupant characteristics, crash factors, or associated injuries identified at autopsy can predict the occurrence or number of ribs fractured.

## Materials and methods

### Crash data—Traffic Accident Reporting System

The crash data used in the present study were extracted from the Traffic Accident Reporting System (TARS). TARS is comprised of all crashes reported to the South Australia Police and is maintained by the Department of Transport and Infrastructure. The study time period was between January 2000 and December 2020. Data from TARS were made available through the Centre for Automotive Safety Research at the University of Adelaide. All cases were motor vehicle occupant fatalities ≥ 18 years of age reported in TARS. Pediatric motor vehicle occupants were excluded as the relative size difference between children and adults can alter the performance and function of seat belts and change the pattern of injury [9, 10]. Furthermore, infants and young children may have a higher injury tolerance to chest impact due to the elasticity of the pediatric thorax [11]. Due to the difference in crash circumstances and therefore patterns of injury, heavy vehicles (e.g., trucks, buses) were excluded from the study. Crash factors selected from TARS for analysis were position in vehicle, restraint status, ejection, crash type, vehicle type, and year of vehicle manufacture at the time of the crash.

### Fatal injury data—coronial autopsy reports

Cases identified in TARS were then cross-matched with corresponding coronial autopsy reports from Forensic Science SA. Occupant characteristics such as body mass index (BMI), cause of death, and the presence of other injuries were extracted from the autopsy reports. Rib fracture information was also extracted from autopsy reports identified through gross examination by the examining pathologist. Concomitant injuries were recorded if the injury description in the autopsy report satisfied the requirements for an Abbreviated Injury Severity score of 3 or above [12]. Individual cases were excluded from the dataset if the pattern of rib fractures were attributed by the examining pathologist to resuscitation attempts or the body had been incinerated.

**Fig. 1:**
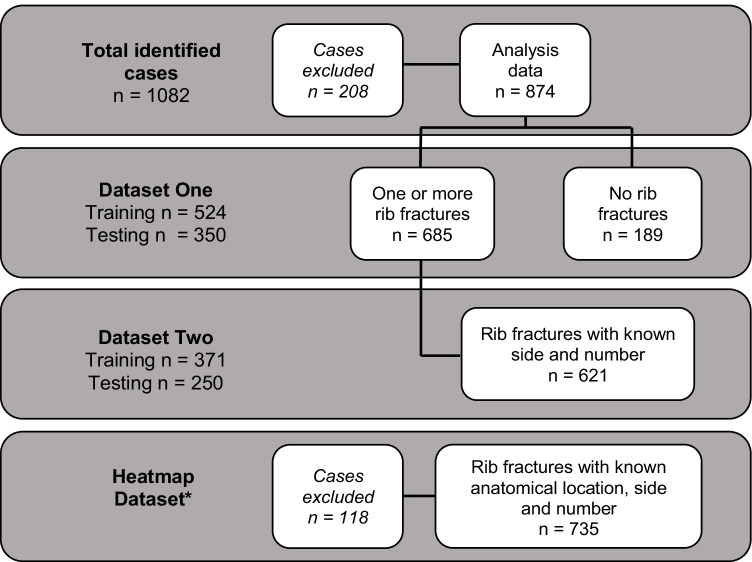
Summary of dataset inclusion and exclusion. All cases were derived from the initial pool of coronial autopsy reports identified with corresponding TARS crash data. Of the 1082 identified cases, 853 cases had rib fractures, 229 cases had no rib fractures. As outlined in the data analysis, cases with missing data are required to be excluded for model development (*n* = 208) however, the development of heat maps permits cases to have missing data. The sample of 735 cases for the heat map dataset was derived from the 853 cases with rib fractures (asterisk symbol). The remaining 118 cases with rib fractures identified in the initial dataset (*n* = 853) were excluded in the final heat map dataset due to missing anatomical, side, or number of ribs fractured

As the coronial autopsy files contained varying levels of detail in regard to rib fracture descriptions, the sample sizes for each level of analysis varied depending on the amount of detail on rib injuries that was available (Fig. 1). Cases with missing data for belt status, seating position, and year of vehicle manufacture were excluded from the logistic regression modelling. However, cases with missing data were still included in the dataset used for heat mapping if data relating to rib number, side, and anatomical location were available (Fig. 1).

### Statistical methods

All analyses were conducted using R studio version 1.4 [13]. Two separate datasets were created for the analyses (Fig. 1). Training (60%) and test (40%) subsets were extracted from the data for individual model development and validation.

Logistic regression modelling was performed (*α* = 0.05) on the training data to predict whether or not a vehicle occupant had sustained at least one rib fracture, including injury type, occupant, and crash characteristics as independent variables. Increasing age is recognised as the strongest predictor for rib fractures and was therefore included in all models as a covariate [6, 14, 15].

The results of the logistic regression are presented as odds ratios (OR) with 95% confidence intervals (CI). Model development was performed using a stepwise method with selection determined using Bayesian information criterion (BIC) scoring. Sternal injury was excluded from the model as sternal fractures rarely occur without rib fractures and therefore behave as a proxy for rib fractures. The testing dataset was then used to assess the fit of the model with performance measured using a confusion matrix.

A linear regression (with *α* = 0.05) was performed to determine if any of the occupant characteristics, crash characteristics, or associated injuries could significantly predict the number of rib fractures (among those who had sustained at least one fractured rib). This linear regression was performed using the training data of dataset two. Results of the linear regression are reported using adjusted *R* squared and *F* statistics with degrees of freedom. All model outputs and goodness of fit tests are included in the Appendix.

Heat maps were generated for selected occupant characteristics, crash factor, and injuries to visualise the anatomical pattern of rib fractures. The proportion of total ribs fractured for the condition of interest was calculated to standardise the appearance of the heat maps across varying sample sizes for each condition.

## Results

A total of 1475 motor vehicle fatalities were recorded in TARS between January 2000 and December 2020, and 1082 coronial autopsy reports were identified that corresponded to TARS fatal crash data. After applying the exclusion criteria involving missing data, 874 cases were included in the analysis (Fig. 1). To summarize the excluded occupant cases, 153 cases had unknown belt status, 12 cases had unknown seating position, 14 cases had indeterminable BMI (due to amputations etc.), and there were 29 cases for which the year of vehicle manufacture was unknown.

### Cause of death

Of the 874 cases, 685 cases had one or more rib fractures. The leading cause of death for those with rib fractures was multiple trauma (54%), followed by head injury (17%) and chest injuries (10%). The remaining 189 cases did not have any rib fractures at autopsy. The main cause of death for occupants without rib fractures was head injury (52%), followed by multiple trauma (20%) and spinal injuries (6.9%). It should be noted that the cause of death was taken as the main injury that resulted in death. Table 1 provides a descriptive summary of the occupant and crash characteristic in fatal cases with and without rib fractures.

**Table 1:** Demographic characteristics of 874 occupant fatalities with and without rib fracture in South Australia (2000–2020)

Characteristic	No rib fracture, *N* = 189	Rib fracture, *N* = 685
Age	32 (18–87)	46 (18–96)
BMI	26 (16–61)	28 (16–57)
Year of vehicle manufacture	1995 (1970–2018)	1996 (1942–2020)
Sex		
Male	147 (78%)	468 (68%)
Female	42 (22%)	217 (32%)
Seat belt		
Not wearing	59 (31%)	201 (29%)
Wearing	130 (69%)	484 (71%)
Ejected from vehicle		
No	159 (84%)	599 (87%)
Yes	30 (16%)	86 (13%)

### Predicting rib fracture patterns

The strongest predictor of one or more rib fractures was increasing age (*p* < 0.001, OR = 1.04, 95% CI = 1.03–1.06). Liver, lung contusion, and hemothorax were also significant factors in predicting one or more rib fractures. Please see appendix for full model output. The accuracy of the model as determined by a confusion matrix was 83% (accuracy = 0.831, 95% CI = 0.788–0.869). The model had sensitivity of 47% (0.474) and specificity of 93% (0.930) when predicting whether an occupant sustained one or more rib fractures.

### Predicting the number of ribs fractured

**Fig. 2:**
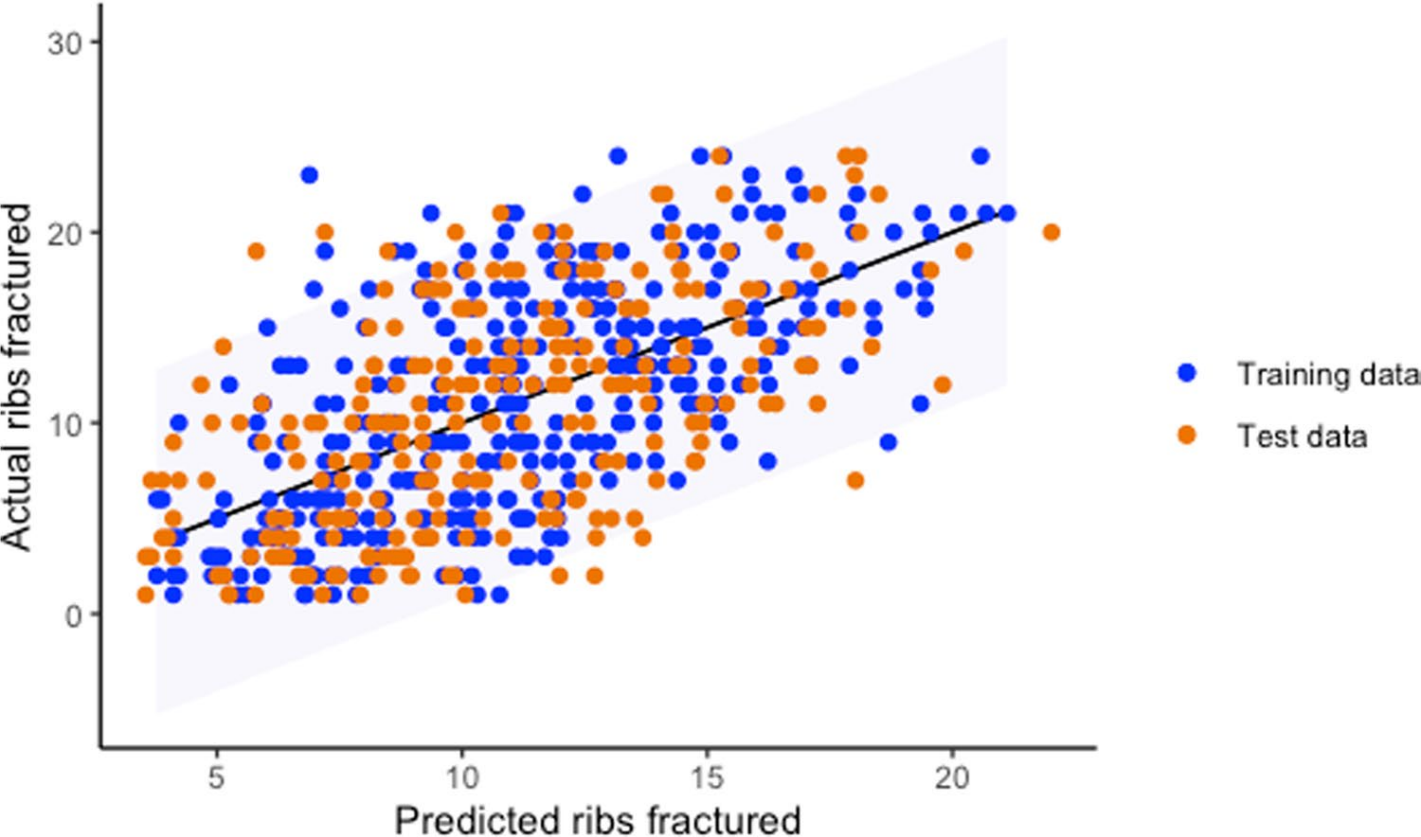
Linear regression of training dataset predicting number of ribs fractured plotted against test dataset. Along the *x*-axis are the values predicted by the linear regression using the training data. The *y*-axis shows the actual values within the test dataset. 95% prediction intervals for number of ribs fractured

Multiple linear regression was used to test if any of the occupant characteristics, crash characteristics, or associated injuries significantly predicted the number of ribs fractured. The overall regression was statistically significant (adjusted *R*^2^ = 0.399, *F* (8,362) = 31.72, *p* < 0.001). The factors found in the regression to be predictive of the number of rib fractures were increasing age (Fig. 2) and the presence of a variety of other injuries including thoracic spinal fracture, lower right extremity fracture, splenic injury, pelvic fracture, aortic injury, lung laceration, and hemothorax. Please see appendix for full model output.

**Fig. 3:**
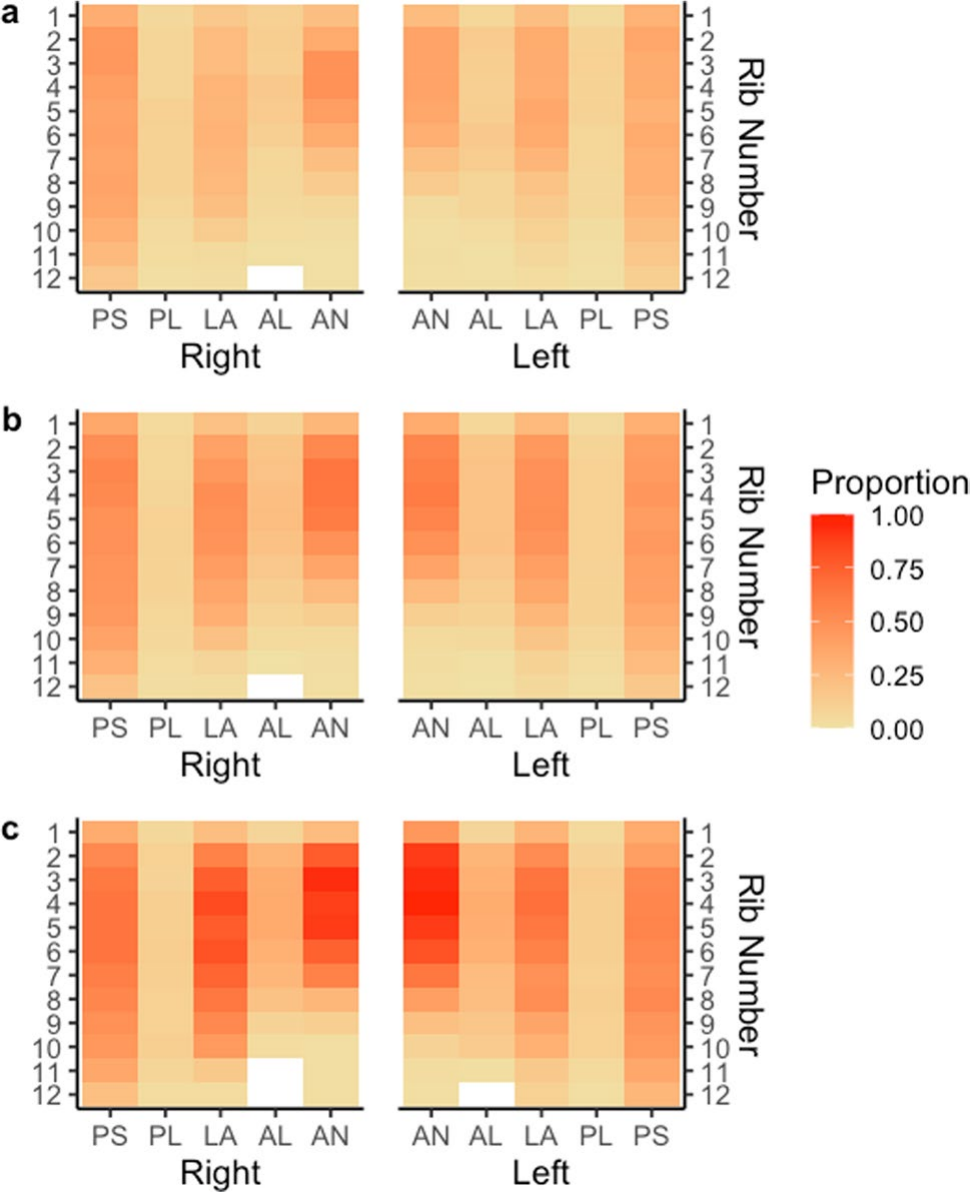
Proportion of rib fractures by rib number, rib cage side, and anatomical location by age. Panel **a** describes the proportion of rib fractures for ages 18–39. Panel **b** describes the proportion of rib fractures for age 40–64. Panel **c** describes the proportion of rib fractures for age > 65 years of age. The abbreviations of anatomical locations are as follows: AN, anterior; AL, anterolateral; L, lateral; PL, posterolateral; PS, posterior

**Fig. 4:**
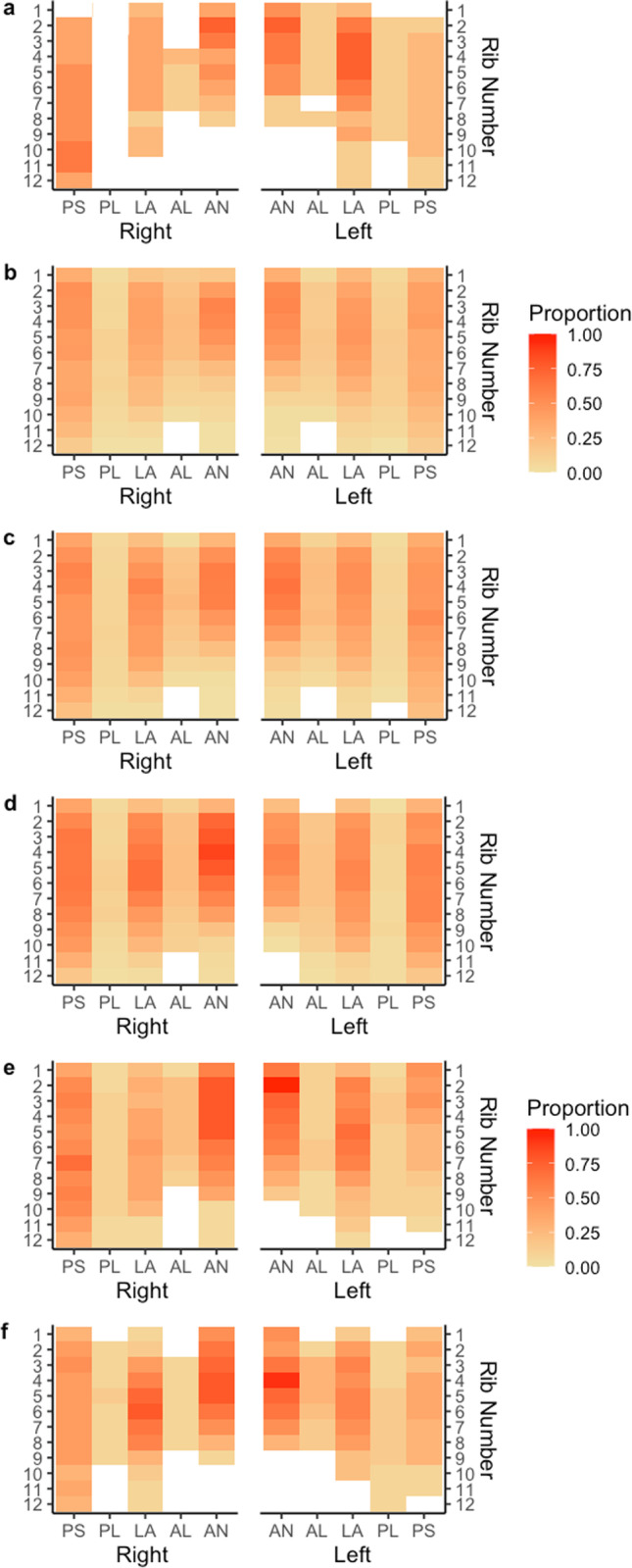
Proportion of rib fractures by rib number, rib cage side, and anatomical location by BMI. Panel **a** describes the proportion of rib fractures for BMI < 17.99. Panel **b** describes the proportion of rib fractures for BMI 18–24.99. Panel **c** describes the proportion of rib fractures for BMI 25–29.99. Panel **d** describes the proportion of rib fractures for BMI 30–34.99. Panel **e** describes the proportion of rib fractures for BMI 35–39.99. Panel **f** describes the proportion of rib fractures for BMI > 40. The abbreviations of anatomical locations are as follows: AN, anterior; AL, anterolateral; L, lateral; PL, posterolateral; PS, posterior

**Fig. 5:**
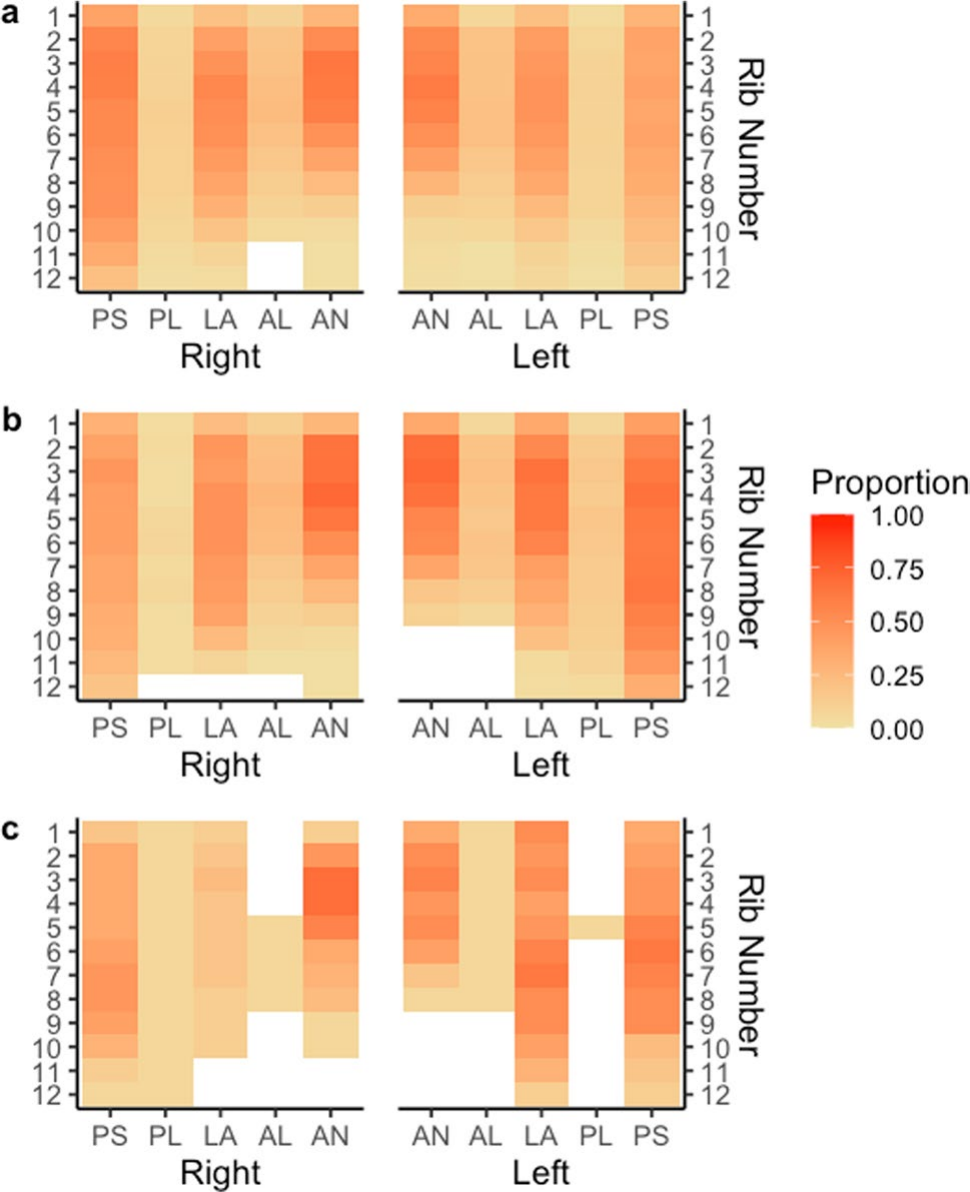
Proportion of rib fractures by rib number, rib cage side, and anatomical location by position in vehicle. Panel **a** describes the proportion of rib fractures for drivers. Panel **b** describes the proportion of rib fractures for front seat passengers. Panel **c** describes the proportion of rib fractures for rear seat passengers. The abbreviations of anatomical locations are as follows: AN, anterior; AL, anterolateral; L, lateral; PL, posterolateral; PS, posterior

**Fig. 6:**
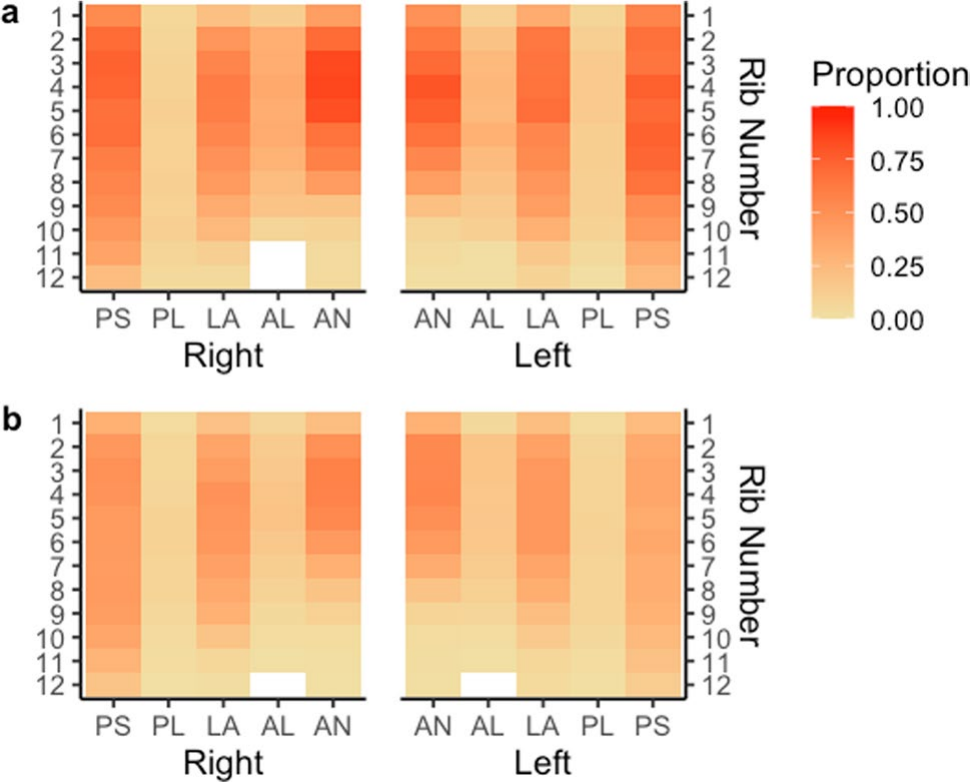
Proportion of rib fractures by rib number, rib cage side, and anatomical location by thoracic spinal fracture. Panel **a** describes the proportion of rib fractures with a thoracic spinal fracture. Panel **b** describes the proportion of rib fractures without a thoracic spinal fracture. The abbreviations of anatomical locations are as follows: AN, anterior; AL, anterolateral; L, lateral; PL, posterolateral; PS, posterior

The following heat maps show the proportion of rib fractures at various anatomical locations on the rib cage for different variable conditions (Figs. 3, 4, 5, and 6).

## Discussion

Increasing age was the strongest predictor of rib fractures as has been noted in previous research [6, 14, 15]. However, the current study demonstrates that the effect of age is stronger than any other occupant or crash characteristic. This effect is clearly shown in Fig. 3 where the proportion of ribs fractured for occupants 65 years and older is far greater than for younger vehicle occupants.

Impact to the thorax results in “posterior displacement of the sternum relative to the spine”, referred to as chest deflection [16]. The number of rib fractures is associated with the magnitude of chest deflection rather than the impact force, and the threshold of injury during chest deflection is dependent on age [16, 17]. Increasing age results in reduced chest deflection tolerance and therefore greater injury at lower magnitudes of deflection [16]. Age-related demineralisation and degradation of cortical bone result in bone remodelling, which causes rib deterioration, while cartilaginous sections of rib undergo calcification and hardening, which reduces rib flexibility. Thus, the ability of the aging ribcage to deflect thoracic impact is reduced and this explains why aged ribs fracture more readily [18].

Increased risk of fatality is associated with increased number of ribs fractured for older occupants. In a study of hospitalised trauma patients, a majority of whom were motor vehicle occupants, Bulger et al. found that, for every additional rib fracture in elderly patients (> 65 years of age), the risk of pneumonia increased by 29% and mortality by 19% [19]. Additionally, rib fractures are frequently associated with other thoracic injuries such as aortic damage and pulmonary contusions. The combined effects of rib fractures and underlying injuries are particularly detrimental to the already reduced physiological reserve and decreased ventilatory capacity of older people. Stiffening of the chest wall, reduced cardiac output, reduced lung capacity, poor blood oxygen exchange, and pain-related limitation of mechanical ventilation in older persons with rib fractures also increase associated mortality [14].

Importantly, restraint use was not predictive of rib fracture or of a greater number of ribs fractured in either model. While seat belts load the thorax during crash impacts, the likelihood of rib fracture is not greater with restraint use compared to no restraint use. Fatalities of seat belt wearers, however, often involve significant impacts that would likely be fatal irrespective of belt wearing [20, 21].

Liver injury was the second strongest predictor of one or more rib fractures. Lung contusion and hemothorax also appeared as significant factors in the model. Similarly, when predicting the number of ribs fractured, fractures of the right leg, splenic injury, and pelvic fracture all appeared more significant than other typical blunt injuries (e.g., aortic injury). The association with non-chest injuries most likely suggests an overall higher level of injury severity. Thoracic spinal fracture is an expected predictor of the number of ribs fractured as the thoracic spine connects to all ribs and, consequently, fracture of one of thoracic vertebrae may produce one or more rib fractures. Figure 6 demonstrates that the posterior anatomical location has a higher proportion of rib fractures for those with a thoracic spine fracture compared to no thoracic spine fracture.

Although linear regression was able to predict the number of ribs fractured, the model had a low predictive power suggesting that there are other factors affecting the number of rib fractures that are not included in this dataset such as speed of impact, vehicle intrusion and point of impact. It may also be the case that the relationship between ribs fractured and injury severity is cumulative whereby the aggregated number of ribs fractured creates distinct levels of injury severity as opposed to the incremental increase in injury severity for every additional rib fractured [22].

While position in a vehicle and BMI were not significant predictors in any of the models, both are well-established modifiers of injury severity and patterns of injury [23–27]. The extremes of BMI (Fig. 4, panels a and f) both have a higher incidence of anterior rib fractures, particularly for rib numbers two to seven. Notably, there were fewer rib fractures in rear-seated passengers (Fig. 5).

There are several limitations to this study, namely the absence of crash data for variables associated with injury severity such as speed of impact, vehicle intrusion, and point of crash impact [28–30]. Other safety devices, such as airbags, are not routinely reported in TARS or coronial autopsy reports and were therefore omitted from the study. Airbags protect the head and thorax during impact, and therefore the extent to which this protective effect is contributing or preventing rib fractures or other injuries remains unclear. Additionally, rib fractures may have been missed during gross examination, predominantly in the posterior anatomical location. Although cases with rib fracture patterns attributable to were removed from the study cohort, it is possible that some of the rib fractures documented at autopsy, particularly those in the anterior anatomical location, were caused by or worsened during resuscitation attempts.

This study demonstrates the effect of increasing age on rib fractures in vehicle crashes, as well as revealing an association with liver injury, pulmonary contusion, and hemothorax. Right leg fractures, splenic injury, and pelvic fractures may reflect the severity of impact.

## Supplementary Information

Below is the link to the electronic supplementary material.

Supplementary file1 (PDF 267 KB)
