# Supplementary material for: Fatal blunt chest trauma: an evaluation of rib fracture patterns and age
Source: Int J Legal Med. 2022 Jul 14;136(5):1351–7. doi: 10.1007/s00414-022-02866-2 (PMC9375745; doi:10.1007/s00414-022-02866-2)
Supplement: Supplementary file 1 — Supplementary file1 (PDF 267 KB) [file 414_2022_2866_MOESM1_ESM.pdf]

Appendix A.

Table A1
Model output predicting one or more rib fractures

Deviance Residuals:

Min	1Q	Median	3Q	Max
-2.9129	0.1188	0.3145	0.5817	1.5832

Coefficients:

	Estimate	Standard Error	z value	Pr(>|z|)
(Intercept)	-1.74721	0.34785	-5.023	5.09e-07 ***
Age	0.04613	0.00808	5.709	1.14e-08 ***
Liver injury (1)	1.42924	0.41109	3.477	0.000508 ***
Lung contusion (1)	0.71049	0.28090	2.529	0.011428*
Haemothorax (1)	2.00830	0.27669	7.258	3.92e-13***

Signif. codes: 0 '***' 0.001 '**' 0.01 '*' 0.05 '.' 0.1 ' ' 1

(Dispersion parameter for binomial family taken to be 1)

Null deviance: 546.37 on 523 degrees of freedom
Residual deviance: 396.98 on 519 degrees of freedom
AIC: 406.98
BIC: 428.29

Number of Fisher Scoring iterations: 6

Table A1 is predicting the probability of rib fracture using the training data from Dataset 1, where the presence of one or more rib fracture was equal to 1.

Table A2

Model output predicting the number of ribs fractured

Deviance Residuals:

Min	1Q	Median	3Q	Max
-9.7630	-3.2974	-0.5812	3.0947	16.1159

Coefficients:

	Estimate	Standard Error	z value	Pr(>|z|)
(Intercept)	1.51507	0.73201	2.070	0.039186 *
Age	0.11278	0.01222	9.231	<2e-16 ***
Thoracic spinal fracture (1)	1.89514	0.60018	3.158	0.001724 **
Lower right extremity fracture (1)	1.48338	0.52424	2.830	0.004921 **
Spleen (1)	1.76754	0.59250	2.983	0.003046 **
Pelvic fracture (1)	1.29633	0.51216	2.531	0.011792 *
Aortic injury (1)	2.09845	0.54559	3.846	0.000142 ***
Lung laceration (1)	1.44577	0.58725	2.462	0.014284 *
Haemothorax (1)	2.52645	0.56345	4.484	9.8e-06 ***

Signif. codes: 0 ‘***’ 0.001 ‘**’ 0.01 ‘*’ 0.05 ‘.’ 0.1 ‘ ’ 1

(Dispersion parameter for binomial family taken to be 1)

Residual standard error: 4.563 on 362 degrees of freedom
Multiple R-squared: 0.4121, Adjusted R-squared: 0.3991
F-statistic: 31.72 on 8 and 362 DF, p value: <2.2e-16

Table A2 is predicting the number of ribs fracture using the training data from Dataset 2.

Plot A1

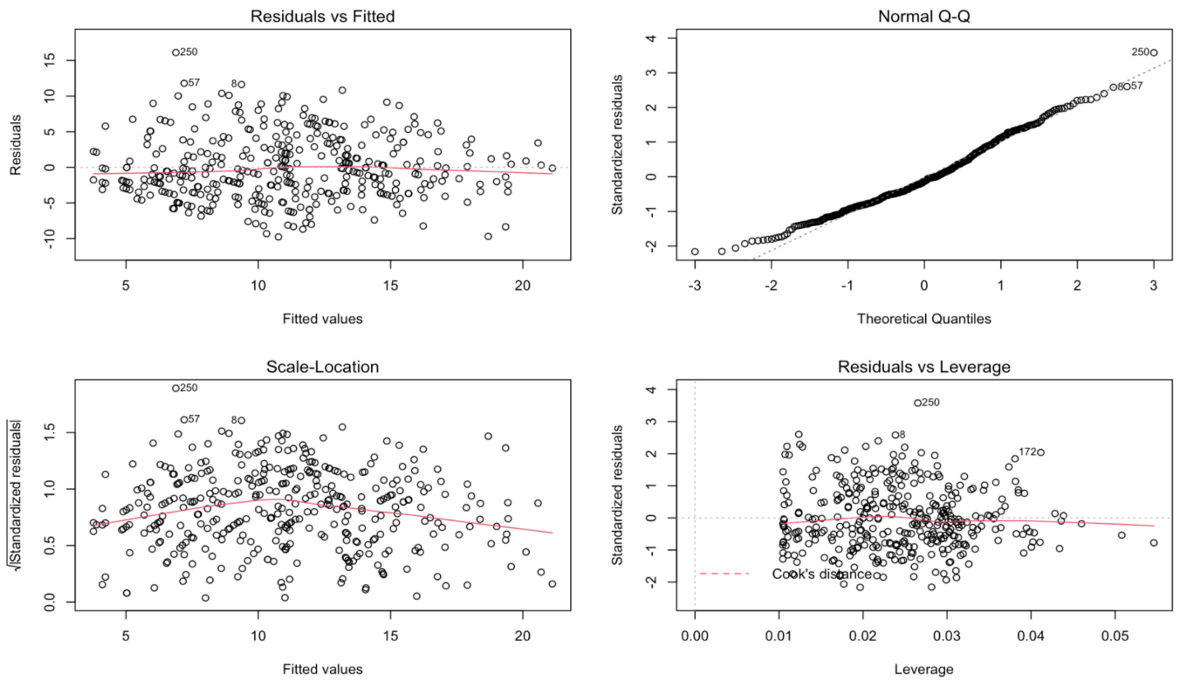

Plot A1 describes the goodness of fit for the linear regression used to predict the number of rib fractures using training data from Dataset 2.
